# Supplementary material for: SlideAudit: A Dataset and Taxonomy for Automated Evaluation of Presentation Slides
Source: Proc ACM Symp User Interface Softw Tech. Author manuscript; Available in PMC 2026 Jun 3. (PMC13227769; doi:10.1145/3746059.3747736)
Supplement: 1 [file NIHMS2175780-supplement-1.pdf]

A Alteration Examples

We provide three examples (Figure 8, 9, 10) from the dataset to showcase different alterations.

B Prompts

We provide different prompts used in SlideAudit evaluations. Each complete prompt used in our evaluation is a combination of the following prompt parts.

B.1 Input Formats

```
You will be provided with an image of a
    presentation slide with bounding boxes
    drawn around various elements.

Analyze this slide for design flaws based on
     the guidance provided.
If you identify a design flaw, specify its
    issue (flaw) name, explanation, and the
    location using normalized coordinates
    (0-1 scale where 0,0 is top-left and 1,1
     is bottom-right).
```

```
You will be provided with two pieces of
    information:
1. An image of a presentation slide with
    bounding boxes drawn around elements and
     labeled with IDs
2. HTML representation of the slide's
    objects with their properties and
    corresponding IDs

When identifying issues, specify its issue
    name, explanation, and the location
    using normalized coordinates (0-1 scale
    where 0,0 is top-left and 1,1 is bottom-
    right).
```

B.2 Evaluation Prompts

```
Please examine the provided slide and
    identify any design flaws or
    deficiencies that negatively impact its
    effectiveness.
```

```
Please examine the provided slide and
    identify any design flaws or
    deficiencies that negatively impact its
    effectiveness.
Consider all aspects of the slide design
    including layout, typography, color,
    visual elements, etc.

IMPORTANT:
- Never identify font-related issues (size,
    family, weight, color, etc.) based on
    missing properties in the HTML
    representation.
- Always verify these properties visually
    from the image. The HTML representation
    is incomplete and should not be used as
    the source of truth for visual
    properties.
- Do not force-find issues when there are
    none. If a slide is well-designed and
    has no issues, return an empty array.

You MUST provide your response following the
     output format. Remember, if you cannot
    find any issues, return an empty array.
```

```
Please examine the provided slide and
    determine if the following specific
    design issues or flaws are present.
Remember to verify all visual properties
    from the image, not from the HTML
    representation.

IMPORTANT:
- For any font-related properties (size,
    family, weight, color, etc.), verify
    them visually from the image.
- Do not identify issues just because of
    missing properties in the HTML
    representation.
- Do not overthink and force-find issues
    when there are none. If this specific
    issue is not present, set issue_present
    to false. We do not want you to over-
    identify issues causing false positives.
- If you cannot find any issues, return an
    empty array.

Potential Categories:
COMPOSITION & LAYOUT RELATED ISSUES:
```

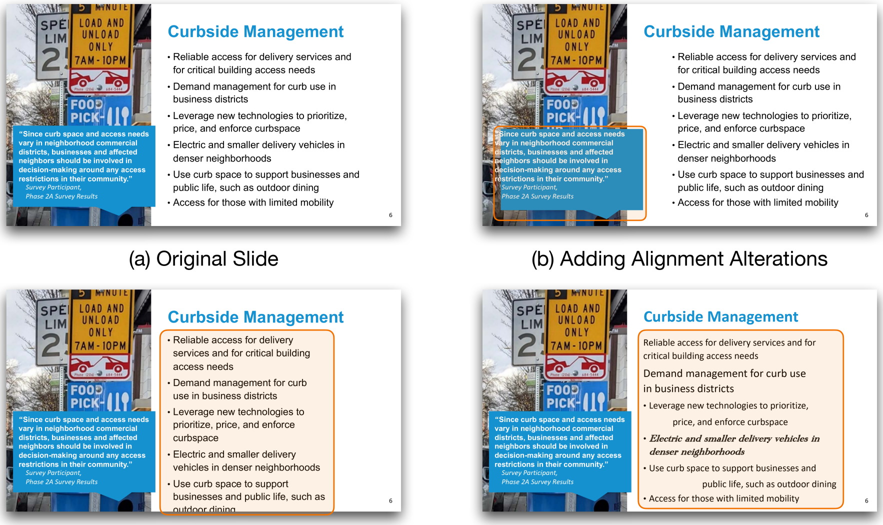

(a) Original Slide (b) Adding Alignment Alterations

(c) Adding Layout Alterations (d) Adding Typography Alterations

Figure 8: SlideAudit dataset alteration example 1.

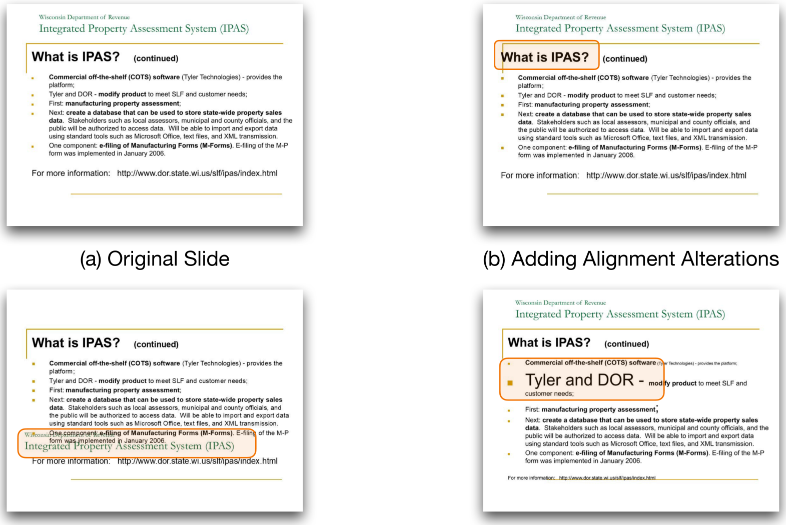

(a) Original Slide (b) Adding Alignment Alterations

(c) Adding Layout Alterations (d) Adding Typography Alterations

Figure 9: SlideAudit dataset alteration example 2.

```
- "Poor Visual Hierarchy" - Elements lack
    clear importance levels, making it
    hard to identify the main point. This
    is not common, because for humans, it
    is easy to identify the main point. Do
     not be strict on this one.
```

```
- "Cluttered Layout" - Too many elements
    crowded together, overwhelming the
    viewer. Distinguish with Excessive
    Text Volume, which is about too much
    text in text boxes. This is for
    general layout.
```

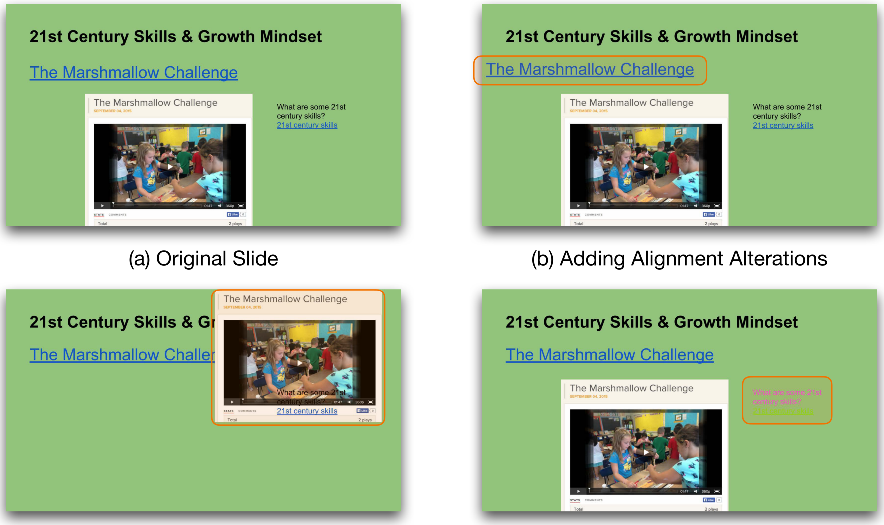

(a) Original Slide (b) Adding Alignment Alterations

(c) Adding Layout Alterations (d) Adding Typography Alterations

Figure 10: SlideAudit dataset alteration example 3.

```
- "Unbalanced Space Distribution or
   Gapping" - Uneven use of space, with
   crowded areas alongside empty ones.
   This is less common, choose with
   caution. Because some space
   distribution is intentional.
- "Object Alignment Issues" - Elements not
    properly aligned with each other.
- "Content Overflow/Cut-off" - Text or
   content extends beyond visible
   boundaries. This is a common issue.
   This is when an object is out of the
   slide boundary, or a text box is cut-
   off too early into lines that are not
   supposed to be cut-off.
- "Occluded Content" - This is very common
   . Choose when some element is blocking
    the view of other elements, even it's
    just a small part of the element.

TYPOGRAPHY ISSUES:
- "Poor Text Hierarchy" - No clear
   distinction between headings,
   subheadings, and body text.  This is
   not common, because for humans, it is
   easy to identify the main point. Do
   not be strict on this one.
- "Illegible Typeface Selection or Usage"
   - Font choice is too decorative or
   complex to read easily.
- "Improper Font Sizing" - Text size is
   small somewhere or large somewhere
   else. Do not choose if the overall
   text size is small or large. Choose
   when there is inconsistent text size
   across the slide.
- "Excessive Text Volume" - Too much text
   instead of concise points. Distinguish
    with Cluttered Layout. This is less
   common, choose with caution. Because
   for humans, they are reading the text
   in their laptop screen.
- "Improper Text Styling" - Inconsistent
   or random use of bold, italics, or
   other formatting. Again, this is only
   for inconsistent text styling over the
    slide. If they are consistent overall
   , even if they are ugly, do not choose
    this.
- "Improper Line/Character Spacing" - Text
    spacing is too tight or too loose.
   Distinguish with excessive text volume
   . A text box can have few text lines
   but still have proper spacing.

COLOR ISSUES:
- "Insufficient Color Contrast for
   Readability" - Text and background
   colors too similar. Not very common.
```

```
  - "Excessive or Inconsistent Color Usage"
      - Too many colors used without clear
      purpose.
  - "Inappropriate or Mismatched Color
      Combinations" - Colors clash or create
       visual strain.

  IMAGERY & VISUALIZATION ISSUES:
  - "Irrelevant Visual Content" - Images don
      't support or relate to the content
      message. Choose with caution because
      people annotated it usually do not pay
       attention to visual content.
  - "Poor Image Quality/Editing" - Images
      are blurry, pixelated, or poorly
      edited.
  - "Improper Image Sizing" - Images sizes
      are either too big or too small.
  - "Inconsistent Visual Style Usage" - (
      This is extremely rare, do not choose
      this unless you are 100 percent sure)
      Mixing different visual styles across
      elements.


{...full_taxonomy_prompting}

Additionally, I will provide you with some
    computational data of this slide for
    more context.
  Remember, they are just references, and
      most times, these contexts are not
      relevant to the issues you are looking
       for.

  I'll provide you with
  (1) a gaze map visualization picture that
      shows where viewers are most likely to
       focus their attention when viewing
      this slide,
  (2) two lists of proximity-based groupings
       and similarity-based groupings of the
       slide's elements, and
  (3) some color metrics for this slide (
      colorfulness, text element contrast
      ratio).

  Here is the grouping analysis data. The
      two lists are groups of close elements
      ' IDs. Each group must have at least 2
       elements. An empty list means no
      groupings are found, which is normal:
  {gaze_data}

  Here is the color data (remember, they are
       not useful for finding issues, just
      for your reference):
  {color_data}
```

B.3 Result Comparison

```
You are tasked with analyzing how AI-
    generated image categories relate to a
    set of 19 predefined categories for
    presentation design issues.

INPUT:
1. PREDEFINED CATEGORIES:
  - There are 19 standard categories for
      presentation analysis:
  {Same categories from full taxonomy
      prompting}

2. AI-GENERATED RESULTS:
  - These are issues independently
      identified by an AI system:
{ai_generated_results}

INSTRUCTIONS:
1. For each of the 19 predefined categories
    above, determine:
  - Whether any AI-identified issues fit
      this predefined category
  - Which specific AI-identified issues best
       match this predefined category

2. For each AI-identified issue, find the
    BEST matching predefined category.
  If the AI-identified issue does not fit
      any predefined category, IGNORE it.

3. Create a comprehensive analysis showing
    how each predefined category relates to
    the AI-identified issues.

IMPORTANT NOTES:
- If an AI-generated category could
    potentially fit multiple predefined
    categories, assign it to the BEST
    matching one.
- If an AI-generated category does not fit
    any predefined category, ignore it.
```

```
- The "present" field should be true ONLY if
     there is at least one AI-generated
    category that fits this predefined
    category.
- Ensure your mappings are logical and
    consistent.
```

C Slide Remediation Examples

We also include examples from the slide remediation study, including generated fix plans in detailed text for a same slide using different LLM variants.

C.1 Example 1

The original slide is shown in Figure 11(1).

Baseline fix plan:


```
[
    {
      "category": "Text Overlap",
      "issue": "The main question text
          overlaps with the subsequent list
          of questions, making it difficult
          to read both sections clearly.",
      "fix_plan": "Separate the main
          question and the list of questions
           by increasing the vertical space
          between them. Adjust the positions
           and ensure that each section has
          sufficient spacing.",
      "expected_improvements": "Improved
          readability and clarity by
          preventing text overlap."
    },
    {
      "category": "Distracting Header
          Background",
      "issue": "The background color of the
          header is bright, which might
          distract readers from the main
          content.",
      "fix_plan": "Change the background
          color of the header to a neutral
          or softer tone that contrasts less
           with the text.",
      "expected_improvements": "Reduced
          distraction and better focus on
          the main content, enhancing
          overall readability."
    }
]
```


Taxonomy-based LLM fix plan:


```
[
    {
      "category": "Cluttered Layout",
      "issue": "The main title overlaps with
           the body text, making the layout
          appear crowded and difficult to
          read.",
      "problem_description": "When text
          elements overlap, it creates a
          visually overwhelming and
          confusing experience for the
          viewer. It becomes difficult to
          discern text hierarchy, making it
          harder to understand the main and
          supporting points.",
      "fix_plan": "1. Increase the spacing
          between the main title and body
          text. \n2. Adjust the size of the
          main title font if necessary to
          create more space. \n3. Consider
          repositioning the main title to
          the top left corner and reducing
          its font size slightly.\n4. Ensure
           there is enough padding around
          the text elements to avoid overlap
          .",
      "expected_improvements": "The slide
          will appear cleaner and more
          organized. Text hierarchy will be
          clearer, and viewers will be able
          to distinguish between the main
          title and supporting content,
          improving readability."
    },
    {
      "category": "Occluded Content",
      "issue": "The body text is partially
          hidden behind the main title,
          making it difficult to read.",
      "problem_description": "When body text
           is occluded, it disrupts the flow
           of information, leading to a poor
           understanding of the content.
          Important details may be missed,
          reducing the effectiveness of the
          presentation.",
```

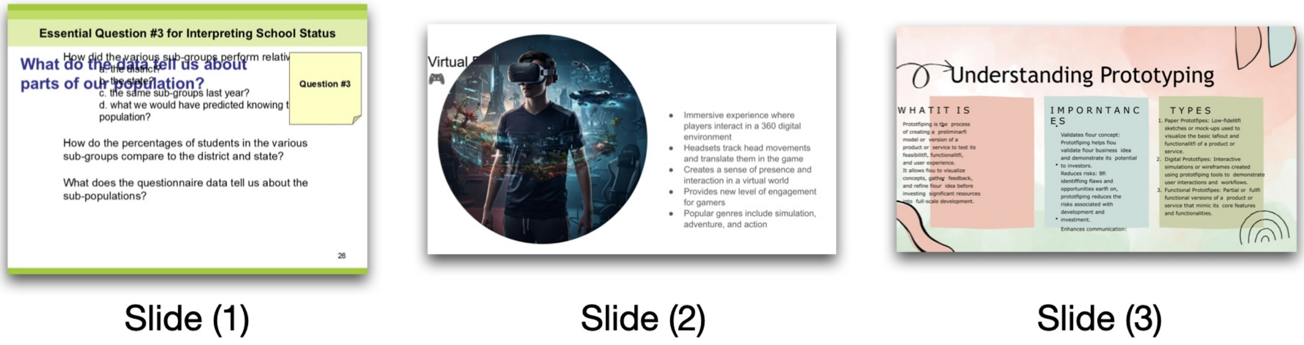

Figure 11: Three example slides from the remediation study.

```
        "fix_plan": "1. Move the body text
            below the main title to prevent
            overlap.\n2. Ensure the body text
            has a clear, distinct area on the
            slide by allocating sufficient
            width and height.\n3. If necessary
            , reduce the font size of the body
             text or break it into bullet
            points to enhance clarity.",
        "expected_improvements": "The content
            will become more accessible and
            easier to read, ensuring the
            audience can fully engage with and
             understand all the information
            presented."
    }
]
```

C.2 Example 2

The original slide is shown in Figure 11(2).

```
Baseline fix plan:

[
    {
        "category": "Text Overlapping with
            Image",
        "issue": "The text \"Virtual Reality
            Gaming\" overlaps with the image,
            making it difficult to read and
            diminishing the visual appeal of
            the slide.",
        "fix_plan": "Move the text box
            containing 'Virtual Reality Gaming
            ' to the right, where it doesn't
            overlap with the image. Adjust the
             font size if necessary to
            maintain balance and readability
            .",
        "expected_improvements": "This will
            increase readability and enhance
            the overall aesthetic of the slide
             by ensuring all elements are
            clearly visible and properly
            aligned."
    },
    {
        "category": "Text Alignment and Size",
        "issue": "The main text box containing
             bullet points starts too far to
            the right, leaving excessive space
             between the image and text.",
        "fix_plan": "Shift the main text box
            to the left, closer to the image.
            Ensure there is consistent padding
             between the text and the image to
             achieve a balanced look. Adjust
            the text box width to ensure all
            content fits properly.",
        "expected_improvements": "The slide
            will appear more balanced, with
            improved flow between the image
            and text. This will make the
            content more engaging and easier
            to follow."
    }
]

Taxonomy-based LLM fix plan:
```

```
[
  {
    "category": "Content Overflow/Cut-off",
    "issue": "The text 'Virtual Reality Gaming' is partially cut off by the left edge of the slide.",
    "problem_description": "When text is cut off, it makes the content difficult to read and unprofessional, negatively impacting the slide's effectiveness and readability.",
    "fix_plan": [
      "1. Select the text box containing 'Virtual Reality Gaming'.",
      "2. Move the text box to the right by adjusting the x-position from 0.0 to a small positive value (e.g., 0.05) to ensure it is fully visible.",
      "3. Ensure the text box width or font size is adjusted if necessary to maintain readability."
    ],
    "expected_improvements": "The text will be fully visible, enhancing readability and professionalism."
  },
  {
    "category": "Occluded Content",
    "issue": "The image is overlapping and partially occludes the text 'Virtual Reality Gaming'.",
    "problem_description": "Overlapping elements can make text unreadable and cluttered, which detracts from the aesthetic appeal and clarity of the slide.",
    "fix_plan": [
      "1. Select the image element.",
      "2. Adjust the x-position of the image from 0.025 to a larger value (e.g., 0.1) to move it slightly to the right.",
      "3. Re-check the position of the text box to ensure no overlap occurs. If overlap still happens, adjust either the image or text box positions further.",
      "4. Optionally, resize the image if space is limited, ensuring it does not compromise the overall layout."
    ],
    "expected_improvements": "The text will no longer be occluded by the image, improving readability and the slide's overall visual balance."
  }
]
```


C.3 Example 3

The original slide is shown in Figure 11(3).

Baseline fix plan:


```
[
  {
    "category": "Spelling Errors",
    "issue": "The text contains misspellings such as 'Prototfiping' instead of 'Prototyping' and 'fiour' instead of 'your'.",
    "fix_plan": "Review and correct all text for spelling errors. Use a spell checker or proofreading tool to ensure accuracy.",
    "expected_improvements": "Improved readability and professionalism of the slide, resulting in clearer communication of key points."
  },
  {
    "category": "Empty Text Boxes",
    "issue": "Some text boxes are empty, indicating possible missing content or placeholders.",
    "fix_plan": "Identify the purpose of each text box and fill them with appropriate content or remove those that are not needed.",
    "expected_improvements": "A complete and polished slide presentation with no unintentional gaps in content."
  },
  {
    "category": "Floating Dots or Small Elements",
```

```
      "issue": "There are very small image
          elements resembling dots that may
          distract viewers.",
      "fix_plan": "Remove or consolidate
          these small elements to maintain
          focus on essential content.",
      "expected_improvements": "A cleaner
          and more aesthetically pleasing
          slide without unnecessary
          distractions."
    },
    {
      "category": "Misalignment of Text
          Blocks",
      "issue": "Text blocks are not well
          aligned, impacting readability and
           aesthetics.",
      "fix_plan": "Align all text boxes
          uniformly. Use alignment guides or
           a grid to ensure consistent
          spacing.",
      "expected_improvements": "Enhanced
          visual balance and easier
          navigation through the slide's
          content."
    }
]
```


Taxonomy-based LLM fix plan:


```
[
    {
      "category": "Object Alignment Issues",
      "issue": "Text headings are not
          properly aligned vertically with
          their associated text boxes.",
      "problem_description": "Misaligned
          headings create a disorganized
          appearance and make it difficult
          for viewers to navigate and
          comprehend the content smoothly.",
      "fix_plan": [
        "Open the slide in a presentation
            editing tool.",
        "Select the text heading 'WHAT IT IS
            '.",
        "Align the heading centrally at the
            top of the associated text box
            .",
        "Repeat for 'IMPORTANCES' and 'TYPES
            ', ensuring vertical alignment
            with their corresponding text
            boxes.",
        "Use consistent spacing and
            alignment guides if available in
             the software."
      ],
      "expected_improvements": "Aligning
          headings will create a more
          coherent and professional
          appearance, improving readability
          and making it easier for the
          audience to follow the information
          ."
    },
    {
      "category": "Content Overflow/Cut-off
          ",
      "issue": "Text content 'IMPORTANCES'
          is cut off within the text box.",
      "problem_description": "Cut-off text
          can cause confusion and prevent
          the message from being fully
          conveyed, reducing the
          effectiveness of the presentation
          .",
      "fix_plan": [
        "Select the text box containing '
            IMPORTANCES'.",
        "Resize the text box to fit all the
            content; increase the height if
            necessary.",
        "Alternatively, adjust the font size
             or line spacing to ensure all
            content is visible without
            overcrowding the text box."
      ],
      "expected_improvements": "Ensuring all
           text is visible will enhance
          understanding and maintain
          audience engagement by allowing
          the full message to be presented
          clearly."
    },
    {
      "category": "Occluded Content",
      "issue": "Decorative elements overlap
          with the text heading '
          Understanding Prototyping'.",
```

```
    "problem_description": "Overlapping
      elements can distract the audience
       and make the heading difficult to
       read, detracting from the overall
       visual impact.",
    "fix_plan": [
      "Select the decorative elements (
         arrows).",
      "Move or resize these elements so
         they do not interfere with the
         text.",
      "Ensure sufficient space is left
         around the heading for clear
         visibility.",
      "Consider changing the layering of
         elements if necessary, sending
         decorative elements to the back
         ."
    ],
    "expected_improvements": "Removing the
       overlap will improve the slide's
      aesthetic and readability,
      ensuring the main heading is clear
       and attention-grabbing."
  }
]
```
